# HTLV-1 in pregnant women from the Southern Bahia, Brazil: a neglected condition despite the high prevalence

**DOI:** 10.1186/1743-422X-11-28

**Published:** 2014-02-13

**Authors:** Marco Antônio Gomes Mello, Aline Ferreira da Conceição, Sandra Mara Bispo Sousa, Luiz Carlos Alcântara, Lauro Juliano Marin, Mônica Regina da Silva Raiol, Ney Boa-Sorte, Lucas Pereira Souza Santos, Maria da Conceição Chagas de Almeida, Tâmara Coutinho Galvão, Raquel Gois Bastos, Noilson Lázaro, Bernardo Galvão-Castro, Sandra Rocha Gadelha

**Affiliations:** 1 LASP/CPqGM/FIOCRUZ, Salvador, Bahia, Brazil; 2 Faculdade de Ilhéus, Ilhéus, Bahia, Brazil; 3 Universidade Estadual de Santa Cruz, Rodovia Ilhéus-Itabuna Km 16–Salobrinho, Ilhéus, Bahia, Brazil; 4 Universidade Estadual do Sudoeste da Bahia, Vitória da Conquista, Bahia, Brazil; 5 LHGB/CPqGM/FIOCRUZ, Salvador, Bahia, Brazil; 6 Escola Bahiana de Medicina e Saúde Pública, Salvador, Bahia, Brazil; 7 LEMB/CPqGM/FIOCRUZ, Salvador, Bahia, Brazil

**Keywords:** HTLV, Bahia-Brazil, Vertical transmission, Pregnant, Prenatal care

## Abstract

**Background:**

As the most frequent pathway of vertical transmission of HTLV-1 is breast-feeding, and considering the higher prevalence in women, it is very important to perform screening examinations for anti-HTLV-1 antibodies as part of routine prenatal care. So far, no studies of HTLV-1 seroprevalence in pregnant women in the Southern region of Bahia, Brazil, have been described.

**Methods:**

Pregnant women were selected at the two regional reference centers for health care from Southern Bahia. A total of 2766 pregnant women attending the antenatal unit between November 2008 and May 2010 have been analyzed. An extra blood sample was drawn during their routine antenatal testing. A standardized questionnaire was applied and all positive plasma samples were tested by ELISA and were confirmed by Western Blot and PCR. Besides that, positive women were contacted and visited. The family members that were present during the visit were asked to be serologically screened to the virus. A prospective study was also carried out and newborns were followed up to two years for evaluation of vertical transmission.

**Results:**

HTLV prevalence was 1.05% (CI 95%: 0.70-1.50). There was no association of HTLV-1 infection with age, education, income and ethnic differences. The association with marital status was borderline (OR = 7.99; 95% CI 1.07-59.3; p = 0.042). In addition, 43 family members of the HTLV-1 seropositive women have been analyzed and specific reactivity was observed in 32.56%, including two children from previous pregnancy.

Conclusion: It is very important to emphasize that the lack of HTLV-1 screening in pregnant women can promote HTLV transmission especially in endemic areas. HTLV screening in this vulnerable population and the promotion of bottle-feeding for children of seropositive mothers could be important cost-effective methods to limit the vertical transmission. Besides that, our data reinforce the need to establish strategies of active surveillance in household and family contacts as important epidemiological surveillance actions for the early detection of virus infection and the prevention of transmission by sexual or and parenteral contact.

## Background

It has recently been estimated that about 5 to 10 million people can be infected worldwide with HTLV [1]. However, as many regions in the world have no data, this value can be underestimated. Nevertheless, Brazil is undoubtedly an endemic area for this virus despite the fact that the worldwide distribution is not homogeneous [2] depending upon the geographic region and the analyzed group [3]. Salvador/Bahia is an area of important prevalence, according to two classical studies: one involving blood donors, and that showed that Salvador has the highest prevalence of Brazil (1.36%) [4] and another involving the overall population from Salvador, which detected a prevalence of 1.76% (reaching 8.4% in women aged 51 years or above) [5]. Besides the higher prevalence in women, the virus was associated with lower education and lower income levels [5], as well as areas with the worst indicators of socioeconomic position [6].

HTLV-1 transmission occurs from mother to child, predominantly through breastfeeding, via sexual intercourse, or through transfusion of cellular blood components [7]. The efficiency of HTLV-1 transmission is related to the route of transmission. The parenteral route (by transfusion or needles sharing) is the most efficient route of transmission. The risk of seroconversion after a transfusion may reach 60% [8]. In the sexual transmission, the infection is more efficient from males to females. In 10 years the risk can be 61% in this direction and only 0.4% in the reverse direction, ie, from women to men [9]. In the vertical transmission, the most frequent pathway of HTLV-1 transmission occurs via breastfeeding, and the risk of infection has been correlated with the provirus load in breastmilk, the concordance of HLA class-I type between mother and child, and the duration of breastfeeding [10-12].

The higher prevalence in women and the possibility of mother-to-child transmission reinforce that it is very important to perform screening for anti-HTLV-1 antibodies during prenatal care and take measures to avoid or, at least, to decrease the risks of transmission. In fact, postnatal infection by breastfeeding seems to play the most important role in vertical transmission; thus, seropostive mothers have been counseled to avoid breastfeeding [13,14]. In Japan, the refraining of breast-feeding conducted by HTLV-1 positive mothers dramatically reduced vertical transmission [14]. In point of fact, it has been suggested that preventing mother-to-child transmission would probably have the most significant impact on the occurrence of HTLV-1-associated diseases [15]. In addition, different clinical manifestation related to HTLV-1, such as: infective dermatitis (IDH), adult T-cell leukemia/lymphoma (ATL), and HTLV-1-associated myelopathy/tropical spastic paraparesis (HAM/TSP) occur in individuals who have been vertically infected [16-18].

In relation to the rate of HTLV-1 infection in pregnant women from Brazil, the prevalence is diverse and heterogenous. In Salvador, Bahia state, it was found 0.84% and 0.88% [18,19], and 0.98% in the town of Cruz das Almas, which is located in the Recôncavo area, 149 Km west from Salvador (4/408) [20]. Nevertheless, these mentioned prevalences are at least three to ten times higher than in other regions of the country [6,21-23].

Ilhéus and Itabuna are the biggest cities in Southern Bahia and are references in health services, treating people from different cities in this region. So far, no studies concerning HTLV-1 seroprevalence amongst pregnant/puerperal women from Southern Bahia have been described. Also considering the importance of these two cities and the high prevalence of HTLV both in Salvador as in other small and mid-sized cities from Bahia, we have decided to evaluate the frequency of this infection amongst women treated at the antenatal units of the two of the largest regional hospitals–one located in Ilheus and the other in Itabuna. In addition, the clinical and epidemiological data of the HTLV-1 positive women were compared with data from a group of HTLV-1 seronegative women. We have also tested HTLV infection in family members of the seropositive women so as to evaluate the possible routes of transmission.

## Results

A total of 2766 pregnant women treated at the antenatal unit between November 2008 and May 2010 were analyzed. Twenty nine pregnant women (1.05%; CI 95%: 0.70-1.50) were HTLV-1 positive, as confirmed by Western blot and PCR. Five of 2766 pregnant women assessed were positive by ELISA and negative after performing the Western Blot, giving a rate of 0.18% of false positive. It was verified one co-infection with HIV and no co-infection with *Treponema pallidum*. Their general prevalences were, respectively, 0.22% (CI 95%: 0.08-0.48) and 0.47% (CI 95%: 0.52-0.80).

All of the 29 HTLV-1 positives were found to be asymptomatic. As regards the place of residence, the city with more cases was Ilheus (n = 14), followed by Itabuna (n = 05). Besides, there have been two cases in Itacaré and one case in the following cities: Coaraci, Wenceslau Guimarães, Uruçuca, Itororó, Camamu, Iguaí, Una and Canavieiras. Amongst the HTLV-positive women, 83.3% reported to be brown, 70.8% were illiterate, and 69% said to receive less than 1 minimum Brazilian wage per month. Only one had received a blood transfusion and, importantly, all HTLV-1 positive women who had another child breastfed him/her. Despite the fact that these women were in the pre-partum room, only two of them have been informed to be HTLV-seropositive during the prenatal care. In these cases, women were advised to have a cesarean delivery and not to breastfeed. Table 1 presents a bivariate analysis for the association of HTLV-1 infection with demographic and social variables. It was found an association with marital status. Yet, the association was not accurate (OR = 7.99; 95% CI 1.08-59.31; p = 0.042). As for the other variables, no statistically significant association has been detected.

**Table 1:** Analysis for HTLV-1 positive and HTLV-1 negative pregnant women

**Variable**	**HTLV-positive N (%)**	**HTLV-negative N (%)**	**Odds ratio**	**95% Confidence interval**
Age (years)				
9-19	04 (16.7)	718 (26.3)	1.00	-
20-29	18 (75.0)	1504 (55.2)	2.15	0.72-6.37
> 30	02 (8.3)	504 (18.5)	0.71	0.12-3.90
Total (N)	24	2726		
Literacity				
Illiterate	17 (70.8)	1771 (64.8)	1.30	0.55-3.20
Literate	07 (29.2)	964 (35.2)	1.00	-
Total (N)	24	2735		
Skin color				
White	02 (8.3)	329 (12.0)	1.00	-
Black	02 (8.3)	687 (25.1)	0.48	0.67-3.41
Brown	20 (83.4)	1623 (59.3)	2.02	0.47-8.71
Yellow	No cases	97 (3.6)	-	-
Total (N)	24	2736		
Income*				
<1.0 mw	20 (69.0)	1773 (65.2)	0.46	0.11-1.85
1-2 mw	03 (10.3)	493 (18.1)	0.85	0.34-2.14
>2 mw	06 (20.7)	455 (16.7)	1.00	-
Total (N)	29	2721		
Marital status				
Married	01 (4.2)	706 (25.8)	1.00	-
Single/Divorced/Widow	23 (95.8)	2031 (74.2)	7.99	1.07-59.3
Total (N)	24	2737		
Stable partner				
Yes	18 (75.0)	2402 (87.8)	1.00	-
No	06 (25.0)	334 (12.2)	2.40	0.94 – 6.08
Total (N)	24	2736		
History of blood transfusion				
Yes	01 (4.2)	93 (3.4)	1.20	0.16-9.24
No	23 (95.8)	2641 (96.6)	1.00	-
Total (N)	24	2734		
Alcohol use				
Yes	04 (16.7)	479 (17.5)	0.94	0.32-2.77
No	20 (83.3)	2258 (82.5)	1.00	-
Total (N)	24	2737		
Smoking				
Yes	01 (4.2)	164 (6.0)	0.68	0.91-5.08
No	23 (95.8)	2573 (94.0)	1.00	-
Total (N)	24	2737		
Tattoo or piercing				
Yes	04 (16.7)	612 (22.4)	0.69	0.23-2.03
No	20 (83.3)	2120 (77.6)	1.00	-
Total (N)	24	2732		

*mw, minimum Brazilian wages per month.

**Figure 1:**
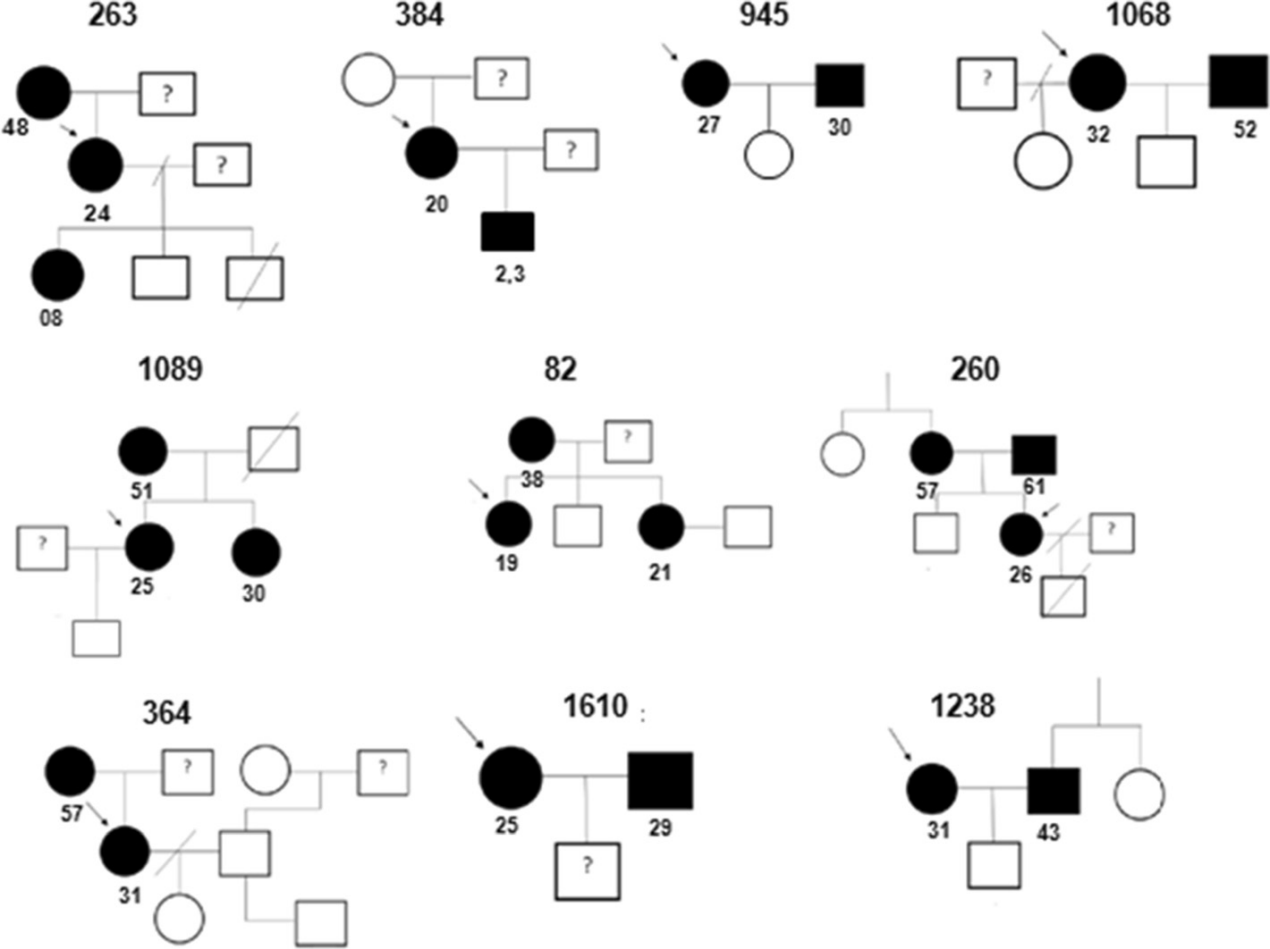
**Pedigrees of ten families who had at least one member infected by HTLV.** HTLV-1-infected (assessed by Western Blot and PCR) and uninfected subjects (tested by ELISA) are shown in black and white, respectively. The arrow indicates the proband in each family. The age in years of each individual HTLV-1 positive is next to their respective symbols.

We have been able to visit 21 women, one of which refused to continue in the study. In this opportunity, samples from 43 family members of the HTLV-1 seropositive women have been collected, including: partner (n = 10), mother (n = 8), father (n = 2), sister (n = 2), brother (n = 2), children of previous pregnancies (n = 15) and others (n = 4). Specific reactivity was observed in 14/43 (≈32.6%) individuals. Among these cases, two were children (1 son and 1 daughter–2.3 and 8 years old, respectively) of previous pregnancies from two HTLV-positive mothers. The other HTLV-seropositives were: 5 mothers, 1 father, 4 partners, 2 sisters (Figure 1). In one case, we had three generations of HTLV-1 infected by the virus (Figure 1). Moreover, half in the evaluated families (10/20) had at least one relative HTLV-1 seropositive.

## Discussion

In this study, the overall analyses of 2766 pregnant women revealed a prevalence of 1.05%. Previous studies in endemic areas of Brazil have found a similar prevalence [19,24] demonstrating that Southern Bahia is another region where the virus circulates with a prevalence much higher than in other regions of the country–at least three to ten times higher [6,21-23]. Additionally, this prevalence was much higher than the prevalence of HIV (0.22%–CI 95%: 0.08-0.48) and *Treponema pallidum* (0.47%–CI 95%: 0.52-0.80) in the analyzed population. It is noteworthy that prevalence rates for these two microorganisms can still be overestimated, since they were calculated from the results of rapid test for HIV and Venereal Disease Research Laboratory (VDRL) for *Treponema pallidum*. In the case of HTLV, all samples were subjected to confirmatory tests.

As we are studying a specific group, the results probably do not represent the overall population. However, in a study comparing data from specific populations (injecting drug users, blood donors and pregnant women), Hlela et al., have suggested that blood donors and pregnant women in Southern America and the Caribbean may be more representative of the general population and can therefore be suitable for estimating prevalence in these regions [25]. In fact, even though the prevalence rates in pregnant women do not represent the overall population, they are very important because the virus can be transmitted to children during pregnancy and, most importantly, during the breastfeeding process. Besides that, different clinical manifestations related to HTLV-1, such as: infective dermatitis (IDH), adult T-cell leukemia/lymphoma (ATL), HTLV-1-associated myelopathy/tropical spastic paraparesis (HAM/TSP) occur in individuals who have been vertically infected [16].

Accordingly, it has been suggested that the detection of HTLV infection through prenatal or neonatal screening can be fundamental in sub-areas with high seropositivity rates, permitting to take preventive measures to reduce vertical transmission [6]. A classical study has demonstrated that the refraining of breast-feeding for HTLV-1 positive mothers has dramatically reduced vertical transmission in Japan [14]. Nonetheless, bottle-fed children can also become vertically infected in much lower frequencies. Then, the prenatal detection is more effective for prevention, whereas additional measures such as an elective cesarean in HTLV-positive pregnants can be taken. In a previous study involving forty-one bottle-fed children from Brazil, no case of vertical transmission was observed. In this case, 81.5% of the children were born by an elective cesarean section and this fact may have contributed to the absence of transmission [26].

In relation of the route of transmission, it was suggested that, in Salvador, the infections have been acquired via breastfeeding, and, in second place, sexually. In this study, the analysis of HTLV-1 serology in relatives, partners and children of previous pregnancies of the index case (pregnant) has revealed HTLV-1 positive cases in different family members, highlighting partners, mothers and children (1 son and 1 daughter–2.3 and 8 years old, respectively). Therefore, it can be assumed that the virus infection in Southern Bahia can be spread both sexually and vertically. In fact, both routes of transmission have been related to HTLV in endemic areas [26]. In addition, it is noteworthy that the two mentioned HTLV-positive children were breastfed. In this study, all of the HTLV-positive women contacted were advised not to breastfeed and the newborns were followed up to two years. Until that time, no positive case has been detected by PCR. Nonetheless, it has been argued that it is necessary to keep in mind that, in developed countries, the advice of not breastfeeding should be made carefully, because the health risk of early weaning can be higher than the risks of HTLV-1 related diseases [27].

Still on the familial transmission, it should be emphasized that half the evaluated families had at least a HTLV-seropositive member. Besides that, the analysis of infection rates in family members indicated a seropositive rate of 32.55% (14/43). This number is higher than that recently found in a survey evaluating familial transmission [28] in Pará state (25.2%), another endemic area for HTLV in Brazil. Without a doubt, the above-mentioned data reinforce the need to establish strategies of active surveillance in household and family contacts as an important epidemiological surveillance action aimed at detecting early the virus infection and preventing the transmission by sexual or parenteral way. In effect, it has been shown that the virus spreads silently within families and that there is a familial aggregation of this infection [28].

This study has detected an association with marital status, but it was not precise. Besides, there was no association of HTLV-1 infection with age, education, and income according to what was found by Magalhães *et al*. [20], in the analysis of pregnant women from a medium sized town in Northern Brazil, unlike the observed in other studies conducted in Salvador, which have found an association between HTLV infection and lower income [5,17]. In the way, we have not found any association between the self-reported skin color and HTLV infection. However, it has been recently detected a higher HTLV prevalence in donors with black skin color [29]. In fact, the two groups (HTLV-1 positive and soronegative) analyzed in our study are very similar in terms of social, demographic and ethnic characteristics, according to the population treated at public hospitals of medium-sized Brazilian cities, where the majority of people is subjected to low levels of income and education.

## Conclusion

In summary, these results are very relevant because: (1) no studies of HTLV-1 seroprevalence in pregnant/puerperal women from the Southern region of Bahia had been described so far; (2) Southern Bahia must be another endemic area for the virus, presenting a high prevalence in pregnant women that is much higher than the national average; (3) the HTLV-1 interfamilial transmission is important and to carry out an active case search can be an important strategy in the epidemiological surveillance of this infection; (4) there is no effective treatment to HTLV infection and interventions to prevent vertical transmission in geographic areas with high prevalence would likely reduce the incidence of mother-to-child transmission and HTLV-related diseases and; (5) last but not least, despite the high prevalence observed in Southern Bahia, it seems that a number of pregnant women treated in the public health system have not been tested to HTLV during the prenatal routine in this region and may have breastfed their babies and could have infected them and spread the infection that reinforce the need for mandatory serological screening in the routine prenatal care of Bahia state.

## Methods

A cross-sectional study involving pregnant women treated at the antenatal units of the two reference health centers from Southern Bahia has been conducted: *Maternidade Santa Helena,* from Ilhéus, and *Hospital Manoel Novaes Santa Casa de Misericórdia*, from Itabuna, between November 2008 and May 2010. This macroregion covers 99 cities, totaling more than 1,025,000 women in childbearing age, and more than 25,000 live births in 2011. The mentioned hospitals treat women from these cities as well as different neighboring cities.

For the study, an extra blood sample was drawn during their routine antenatal (syphilis, HIV, ABO and Rhesus) appointments. A standardized questionnaire has been applied after informed consent to collect the following data: age, formal education, history of smoking, alcohol consumption, blood transfusion, past medical history, current medication, and income level. Furthermore, the medical records were analysed to know the results of two tests: (1) rapid test for HIV and (2) VDRL. It was included in the analyses all women who fulfilled the following criteria: (1) collected blood sample in the antenatal unit; (2) answered the standardized questionnaire; (3) signed the consent informed (in younger than 18 years old, the parents/legal guardians were asked for the permission). The project was approved by the Ethic Committee of *Universidade Estadual de Santa Cruz* (UESC).

HTLV-1 infection was assessed by ELISA (Ortho HTLV-I/HTLV-II Ab-Capture ELISE Test System). The positive plasma samples were tested repeatedly in duplicate and confirmed by Western blot (HTLV BLOT 2.4–Genelab Diagnostic). Besides, the DNA of 29 HTLV-1 positive samples was extracted by QIAgen Kit (QIAamp DNA Blood Kit), followed by a nested-PCR for the Long Terminal Repeat (LTR) region on HTLV-1. Two HTLV-1 overlapping fragments were amplified: a LTR-gag 473 bp and a LTR-tax 479 bp, as previously described [30]. HTLV-positive women were contacted by phone and visited by a healthcare team, which included, among other professionals, a pediatrician. In this opportunity, the results and the HTLV condition have been explained, and samples of family members (spouse, partners children or others, considering the known transmission routes for the virus) have been collected after informed consent to analyze HTLV infection and interfamilial transmission. All those who had interest in performing the diagnosis of HTLV were include. For minors, parents or guardians were consulted and should authorize the test. Besides, a prospective study was also carried out and newborns were followed up to two years for evaluation of vertical transmission.

Frequency distributions were determined for each variable. Age was examined in 3 different strata to analyze trends in prevalence (9-19 y; 20-29 y and 30 y or more). The other variables (and no exposure condition) were: education (illiterate or literate), income (<1.0 minimum Brazilian wages per month (mw); 1-2 mw and >2 mw), ethnic classification, and marital status (married/with partner or single/Divorced/Widow). Known risk factors (previous blood transfusion, have tattoos and/or piercings) and lifestyle habits have been analyzed (alcohol consumption and smoking). A bivariate analysis was carried out.

Odds Ratio (ORs) and 95% confidence intervals (95%CIs) were calculated to measure the association of selected variables with HTLV-1 infection. The statistical package STATA version 10.0 was used for statistical analyses. P-values < 0.05 were considered significant.

## Competing interests

The authors declare that they have no competing interests.

## Authors’ contributions

MAGM: participated in the design of the study, carried out the PCR, carried out the maintenance of the database and helped to draft the manuscript. LPSS, TCG, RGB, AFC: applied the standardized questionnaire, the informed consent and processed the blood samples to perform serology. NBS, MCCA: participated in the design of the study, performed the statistical analysis and helped to draft the manuscript. LCA: participated in the design of the study and helped in the realization of molecular analyzes. BGC: participated in the design of the study, helped to draft the manuscript. LJM, SMBS: participated in the design of the study, helped in identifying positive women, made contact and scheduled the visits, and helped to draft the manuscript. NL: carried out the ELISA and Westen Blot and carried out the blood collection during the visits. MRSR: participated in the design of the study and provided orientation concerning the result of the tests and about breastfeeding during the project. SRG: conceived of the study, coordinated it and drafted the manuscript. All authors read and approved the final manuscript.
